# Lip adhesion revisited: A technical note with review of literature

**DOI:** 10.4103/0970-0358.59283

**Published:** 2009

**Authors:** Krisztián Nagy, Maurice Y. Mommaerts

**Affiliations:** Bruges Cleft and Craniofacial Centre (Director: M. Y. Mommaerts), General Hospital St. Jan, Bruges, Belgium

**Keywords:** Cleft lip, lip adhesion, large clefts

## Abstract

**Context (Background):**

Lip adhesion is a direct edge approximation without changing lip landmarks or disturbing tissue required for definitive closure. This converts a complete cleft into an incomplete cleft, facilitating and enhancing subsequent definitive lip and nose repair.

**Aim:**

The study aims to describe our technique of lip adhesion and its morbidity, and discuss the rationale for its use.

**Settings and Design:**

Retrospective follow-up study of complete clefts operated upon in the Bruges Cleft and Craniofacial Centre, at the supra regional teaching hospital AZ St. Jan, Bruges, between June 1, 1991 and May 1, 2009.

**Methods and Material:**

The group comprised 33 unilateral and 24 bilateral lip adhesion procedures. The medical files were reviewed for changes in surgical technique, morbidity, and complications and their treatment.

**Results:**

The lip adhesion procedure was performed at the age of two to eight weeks postnatal, and definitive lip closure, at the age of four to six months. In all cases, segment repositioning was further controlled by a palatal guidance plate. Wound dehiscence occurred in eight patients (14.0%), and three patients (5.3%) required reoperation.

**Conclusions:**

Although complications occurred, the beneficial effects of lip adhesion in combination with a guidance plate outweighed the risks for anatomical reconstruction of a platform for definitive lip and nose repair. Modifications are suggested to reduce these complications.

## INTRODUCTION

Lip adhesion is a preliminary edge approximation, not disturbing tissue necessary for definitive cleft lip repair.[1] The principle is to convert a complete cleft into an incomplete one. The manoeuvre facilitates definitive closure[2–7] reduces the lip/nasal deformity by moulding of the maxillary segments[1,8] and facilitates feeding.[1,8]

However, it also adds another operation with its associated complications and expenses[4–5,7,9] and it is technically demanding because of limited tissue quantity.[4–5,7] It could also destroy tissue valuable for definitive repair.[1,4–5,9]

We describe our technique with modifications based on the morbidity observed in our series.

## MATERIAL AND METHODS

The medical files of patients with primary complete unilateral and bilateral cleft lip with or without cleft palate, treated between June 1, 1991 and May 1, 2009, were retrieved, and collected data were analyzed.

**Figure 1:**
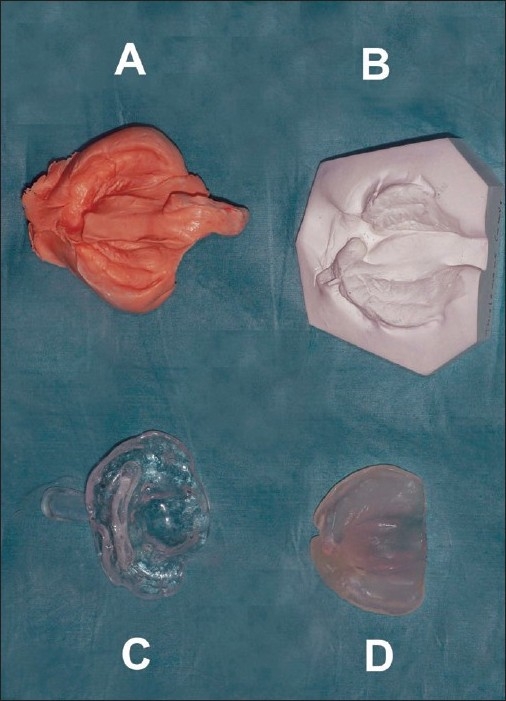
Instrumentarium for guidance plate preparation, (A) Silicone impression material in an individual impression tray, (B) Plaster model of the upper jaw, (C) An individual impression tray, (D) The guidance plate

**Figure 2:**
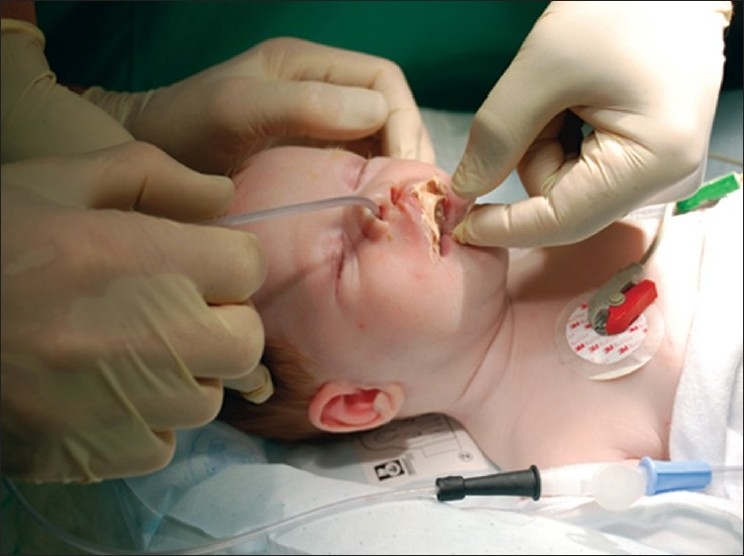
Impression-taking procedure under general anaesthesia using an open system. A gastric tube is placed intranasally into the hypopharynx. Sevoflurane and oxygen are given through the tube, and this gas mixture is exhaled by the patient through the anatomic airway.

**Figure 3:**
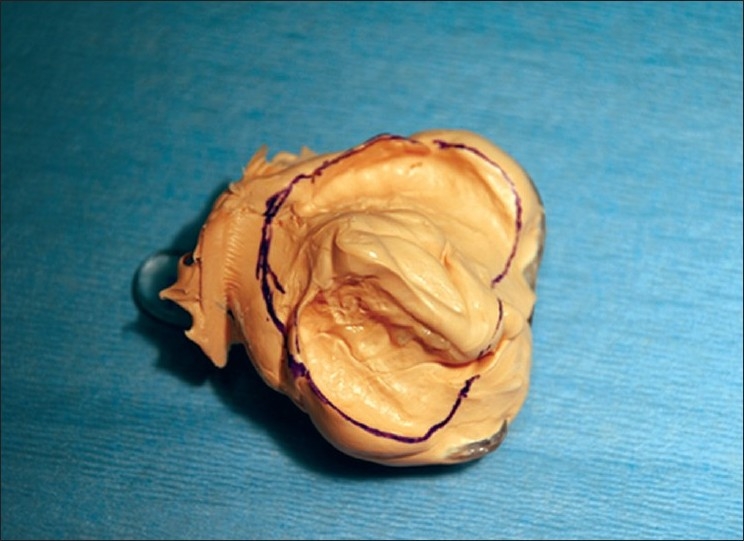
Silicone impression material of the upper jaw with marking of the extension of the palatal guidance plate

The early lip segment and nasal platform management of complete clefts begins with a lip adhesion when the patient is aged three to four weeks. Two weeks later, impressions are taken, and a maxillary guidance plate in soft and hard acrylic is fabricated in a dental laboratory. From 1991 until 2006, alginate impression material (Cavex CA37, Cavex Holland BV, The Netherlands) was used for this purpose but since 2006, silicone impression material (Alginot, Kerr, Romulus, MI, USA) was used. The impression tray is individually prepared from the impression taken at the time of the lip adhesion [Figure 1]. The impression is taken under general anaesthesia using an open system [Figure 2]. The extension of the palatal guidance plate is marked on the impression, according to the clinical situation [Figure 3].

The plate is adjusted every two to three weeks to move the segments into a more normal position. Essentially, the smaller segment is guided forward, and its anterior edge is guided outward. The anterior part of the greater segment is rotated inward. The palatal shelves are guided into a horizontal location by keeping the tongue out of the cleft palate and by grinding the plate down lateral to the septal border. The alveolar cleft is not necessarily closed. On the contrary, the lesser segment is placed into normal relation with the lower arch and often needs expansion. Hence, true alveolar hypoplasia and associated defect(s) become visible.[10–11]

All patients are given intravenous antibiotics preoperatively and postoperatively. The nasal retainer is removed seven to 10 days after surgery during an outpatient consultation. Definitive lip closure is performed at a second procedure.

A modified Millard rotation-advancement technique was used in the first years; in later series, the technique according to Mohler[12] and Asencio[13] was used for closure of unilateral cleft lips, and a Millard-Mulliken (phase III)[14] technique was used for closure of bilateral cleft lips when the patient was at the age of four to six months.

### Surgical Technique of Lip Adhesion

The procedure is performed under anaesthesia with oral intubation. The cleft edge incisions for the adhesion are marked, staying well away from the landmarks of definitive repair. The rectangular flaps are both mucosal-based, and they extended from the nasal base as far caudally as the markings of the definitive lip incisions [Figures 4A–B and Figure 5A]. The operation preferentially starts at the lateral segment. The mucosal flap is elevated submucosally. The preparation proceeds further, under the orbicularis oris muscle fibres, in the direction of the alar base.

**Figure 4A:**
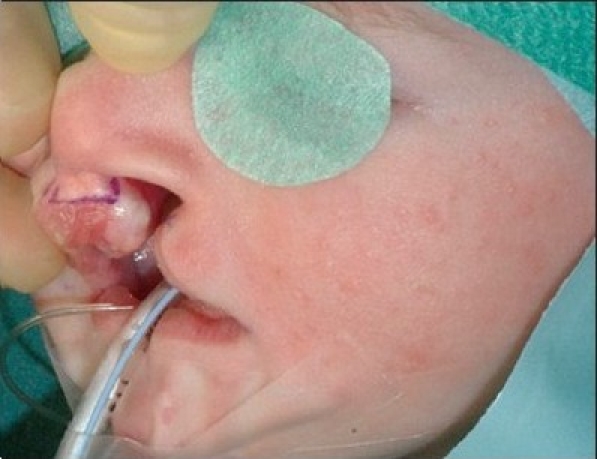
Step-by-step documentation of the surgical technique of unilateral lip adhesion Incision is marked on the medial cleft edge.

**Figure 4B:**
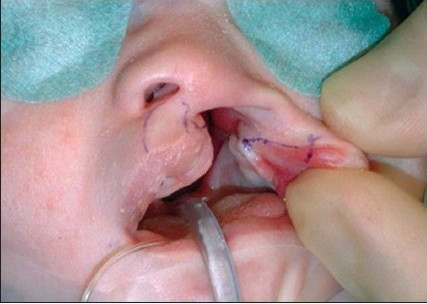
The incision is marked on the lateral cleft edge.

**Figure 5A:**
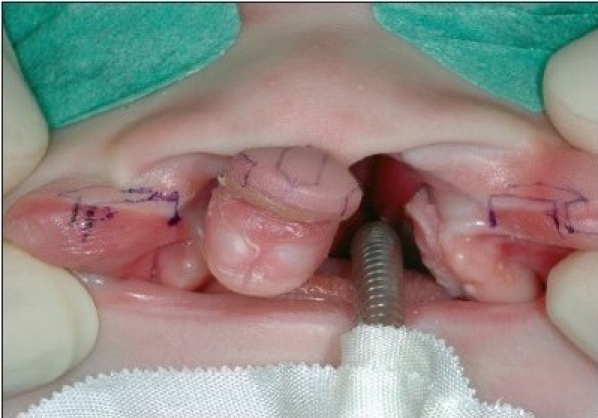
Step-by-step documentation of the surgical technique of bilateral lip adhesion. (Incisions of rectangular mucosal flaps and those for the subsequent definitive closure are marked on the cleft edges)

At the alar base, dissection continues supraperiosteally. We consider it mandatory to release the paranasal musculature at the piriform aperture to enable tension-free approximation. In the medial lip segment, the mucosal flap is elevated as in the lateral part. Supraperiosteal dissection in the medial part proceeds to the septal base. In case of apparent septal dislocation, the caudal septum is released. The rectangular mucosal flaps are approximated with 5-0 polyglactin 910 suture (Vicryl, Ethicon, Neuilly, France) [Figures 4C and 5B].

**Figure 4C:**
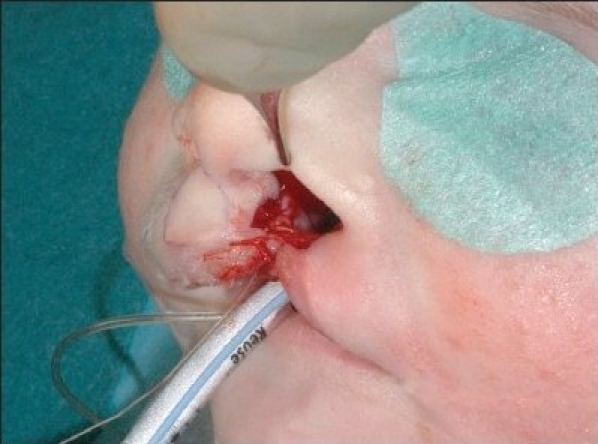
The mucosal flaps are sutured.

**Figure 5B:**
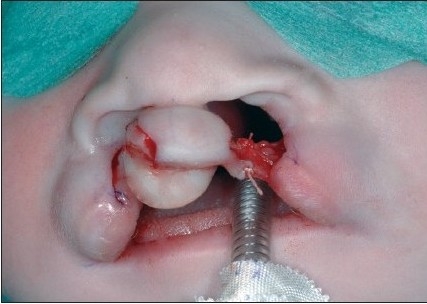
The mucosal flaps elevated on the right side and sutured on the left side

A 4-0 polydioxane suture (PDS, Ethicon) is passed at the level of the alar base bilaterally. The suture begins in the cranial extension of the lateral lip wound, continues under the muscle bundles to the alar base, and pierces the skin. The needle re-enters the skin perforation and continues at a subcutaneous level, above the muscle bundles. The needle enters the cranial end of the medial wound and passes further subcutaneously under the nasal spine until the contra lateral alar base is reached. It pierces the skin again, re-enters the perforation, and continues submucosally to the cranial end of the medial wound [Figure 4D and Figure 5C]. The suture is clamped. Septal correction is performed by attaching the caudal septum to the lateral lip muscles with a 4-0 polydioxane suture (PDS, Ethicon). A second 4-0 polydioxane suture (PDS, Ethicon) is placed at the mid-level of the orbicularis oris muscle, in the same way as the previous suture, and the suture is clamped. The last 4-0 polydioxane suture (PDS, Ethicon) is placed in the same way at the level of the vermilion-skin junction [Figure 4E and Figure 5D]. At this point, all three knots are tied, with special attention to achieving good approximation and closure of the nostril base [Figure 5E].

**Figure 4D:**
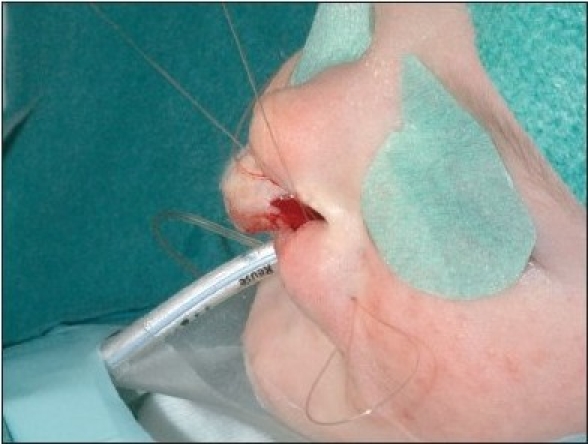
The polydioxane suture is passed at the alar base.

**Figure 5C:**
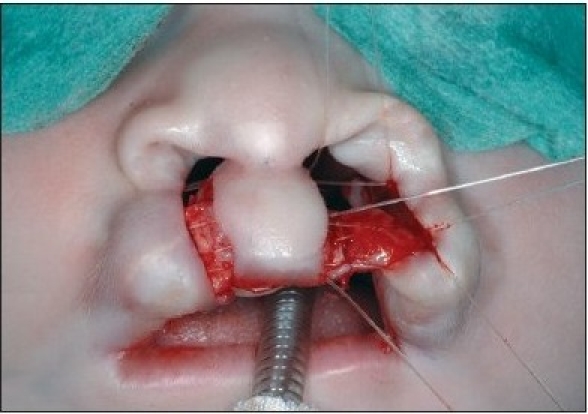
The polydioxane suture passed at the alar base

**Figure 4E:**
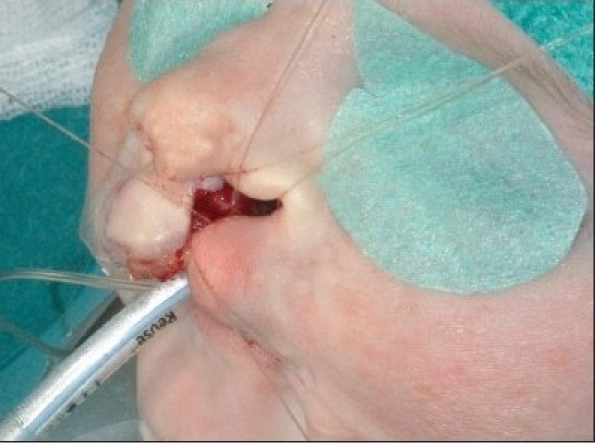
Shows polydioxane sutures approximate the cleft edges at three levels

**Figure 5D:**
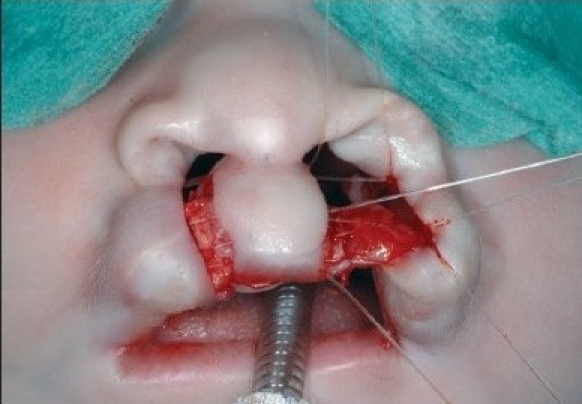
The polydioxane sutures placed at three levels in the lip

**Figure 5E:**
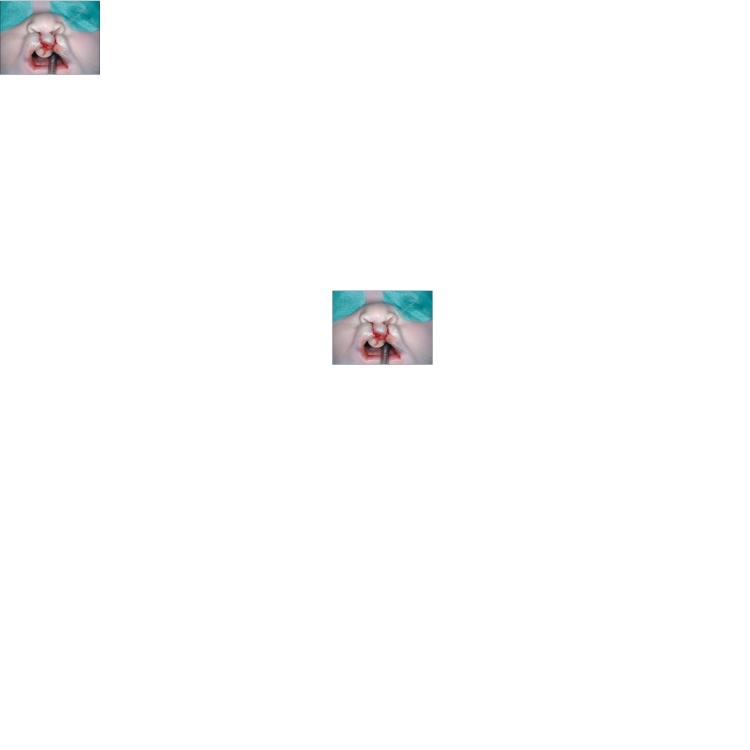
The polydioxane sutures approximate the cleft edges

After approximation of the muscle stumps, the skin is closed without tension with everting stitches of 5-0 polyglactin 910 suture (Vicryl, Ethicon) [Figures 4F and 5F]. An individual quilting stitch of 3-0 polyglactin 910 suture (Vicryl, Ethicon) closes the nasal floor and reduces dead space in the base of the wound funnel. The suture is placed at the lateral nasal vestibule, pierces the lateral mucosal flap, re-enters the medial mucosal flap, and appears through the skin of the medial nasal vestibule [Figure 5G]. This horizontal mattress stitch closes the funnel, which could otherwise entrap nasal mucus, saliva, and milk.

**Figure 4F:**
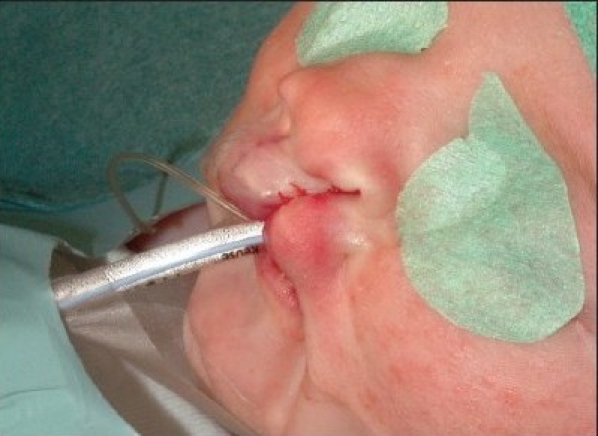
Shows the skin closed with everting stitches of polyglactin 910 suture

**Figure 5F:**
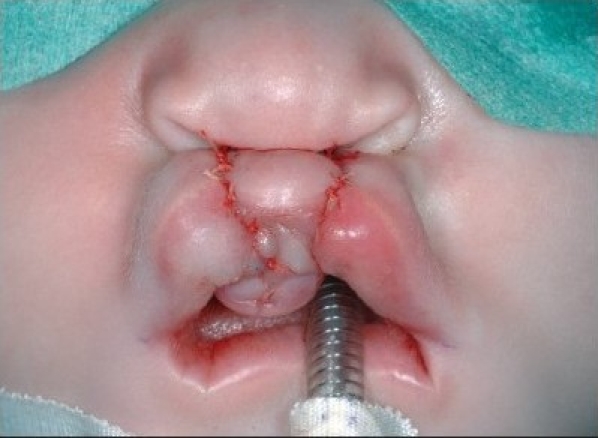
Shows the skin closed with everting stitches of polyglactin 910 suture

**Figure 5G:**
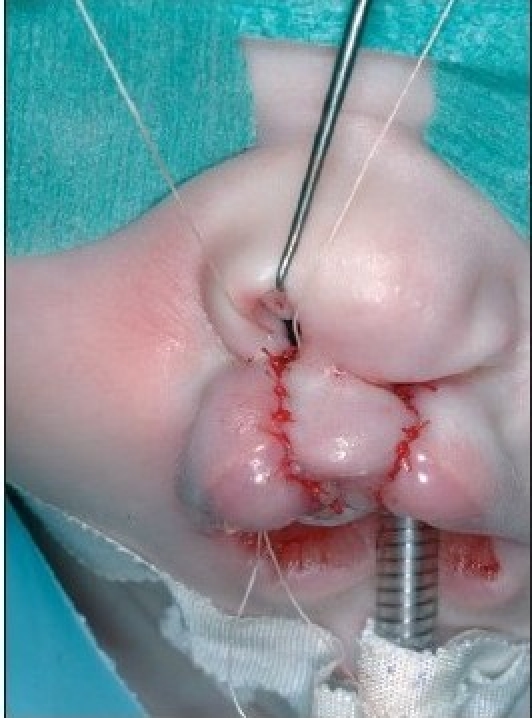
Shows a quilting stitch of polyglactin 910 suture used at the nasal floor to reduce dead space

A short nostril retainer (Koken, Tokyo, Japan) is placed to keep the nasal airway patent. This is fixed to the lateral nasal vestibule by a quilting stitch of 3-0 polyglactin 910 suture (Vicryl, Ethicon), which re-enters the skin at the site of the suture channel to prevent scarring [Figure 4G]. A Logan bow is individually bent and fitted with adhesive tapes (Suture-Strip, Derma Science, Toronto, Canada) to release tension from the lip sutures [Figure 4H and Figure 5H].

**Figure 4G:**
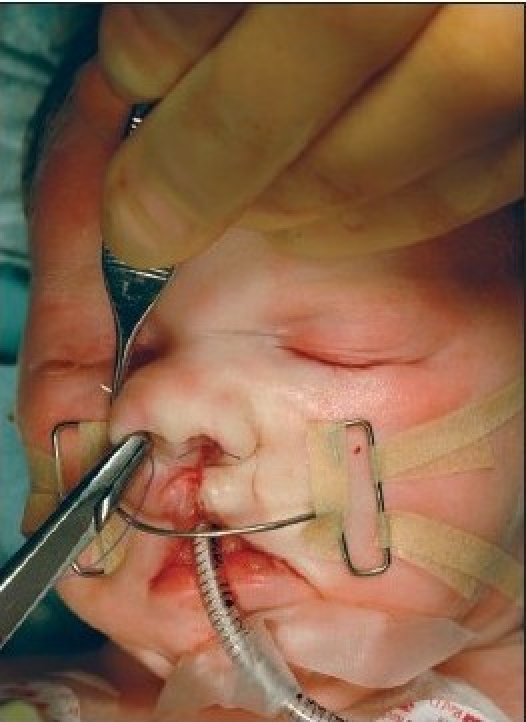
Shows the nostril retainer fixed to the lateral nasal vestibule by a quilting stitch of polyglactin 910 suture

**Figure 4H:**
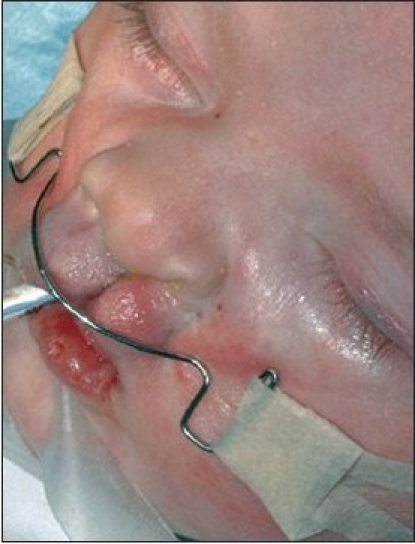
Iimmediate postoperative situation with a Logan bow fixed by adhesive tape

**Figure 5H:**
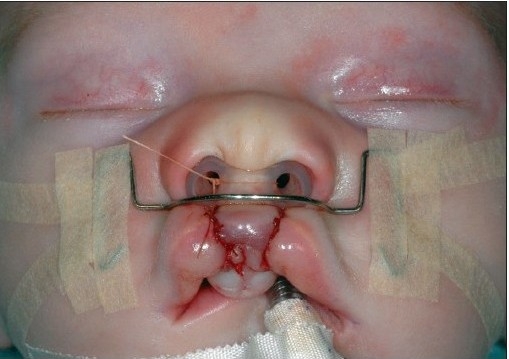
The immediate postoperative situation with the nostril retainer fixed by polyglactin 910 suture and the Logan bow fixed by adhesive tape

**Figure 6:**
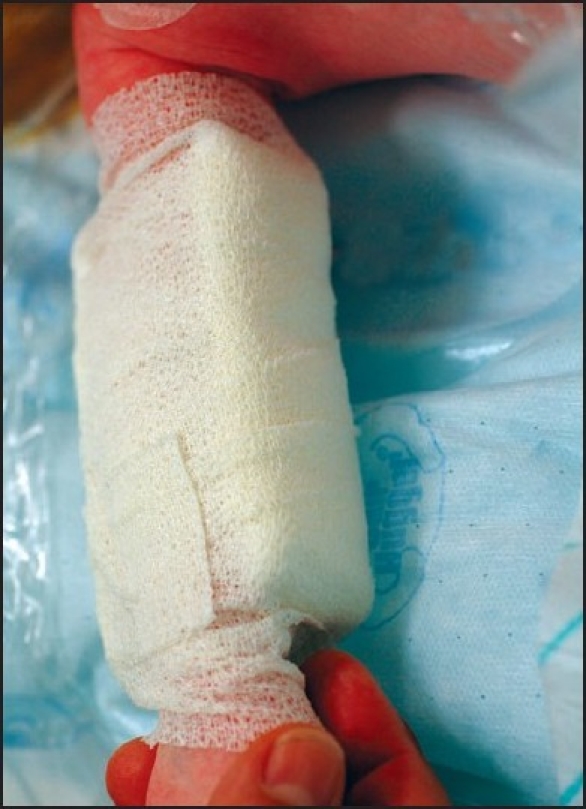
Elbow retainers in place to prevent unwanted manipulation by the infant

The wound is covered with an ointment containing antibiotics, steroids, and pure petrolatum jelly to facilitate wound healing. Long-acting local anaesthetic solution (7,5% ropivacaine hydrochloride, Naropin, Astra Zeneca, Bruxelles, Belgium) is injected infraorbitally at the cleft side(s). Elbow restraints are used to prevent unwanted manipulation by the infant [Figure 6].

### Modifications in surgical technique

The described technique represents our current clinical practice, as a result of several modifications due to clinical experiences.

1. From 1991 until 2004, on one side of the cleft, a mucosal-based rectangular flap and on the other side, a skin-based rectangular flap was elevated. These flaps were approximated like hinge-door flaps. Since 2004, we elevate only orally based flaps, because the mucosal membrane is less stretchable than the skin since it is tethered by the gingiva.
2. Since 2006, a tumescent dose of lidocaine HCl 1% with epinephrine 1:80000 was injected into the lip and nasal base for haemostasis.
3. For muscle closure, 4-0 polyglactin 910 (Vicryl, Ethicon) was used initially, but since 1994, we have used 4-0 polydioxane (PDS, Ethicon). Resorption of polydioxane takes more time, preventing dehiscences and facilitating soft tissue moulding effect on maxillary segments.
4. Since1995, polydioxane suture is used at three levels instead of two, to cope with the tension.
5. Initially, skin was closed with 5-0 nylon (Ethilon, Ethicon), which has been replaced by 5-0 polyglactin 910 (Vicryl, Ethicon) since 1995, making suture removal unnecessary.
6. The nostrils were packed with gauze impregnated by antibiotics (Terra-Cortril, Pfizer, NY, USA) until 1994, to prevent wound inflammation. Since then, a nostril retainer (Koken, Tokyo, Japan) was used.

## RESULTS

The group comprised 57 consecutive patients, 18 girls and 39 boys [Table 1].

**Table 1:** Demography of Our Patient Series (Data are presented as number of patients) (%)

Unilateral cleft lip	2 (3.5%)
Unilateral cleft lip and palate	31 (54.4%)
Bilateral cleft lip	2 (3.5%)
Bilateral cleft lip and palate	22 (38.6%)
Sex:	
Male	39 (68.4%)
Female	18 (31.6%)

Lip adhesion was performed in complete unilateral and bilateral cleft patients at the age of two to eight weeks. The delay to eight weeks of age was due to premature birth in some cases. Definitive closure was performed at the age of four to six months. In all 57 cases, the standard definitive closure technique was applied, and there was no need for modification to address extreme anatomical situations. Large amounts of supraperiosteal undermining of the soft tissues were not necessary in any case [Table 2].

**Table 2:** Timing of Operative Procedures (Data are presented as mean ± standard deviation)

Age at lip/nose adhesion	39 ± 22 days (5.6 weeks)
Age at definitive closure	155 ± 42 days (5.2 months)
Time between lip adhesion and definitive closure	116 ± 33 days (3.9 months)

**Figure 7:**
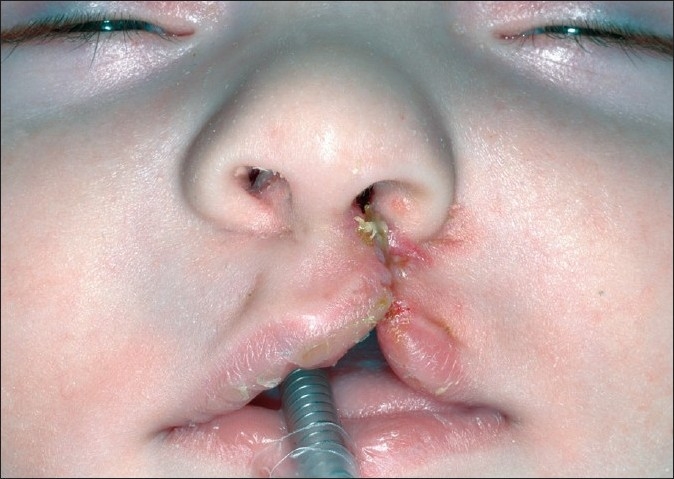
Partial dehiscence six weeks after lip adhesion

Eight patients (14.0%), seven with unilateral and one with bilateral clefts, all male patients, had postoperative dehiscence [Figure 7]. Three patients (5.3%) required readmission for reoperation, and these secondary operations were successful in each case. In three patients (5.3%), postoperative inflammatory reaction was treated with oral antibiotics [Table 3].

**Table 3:** Rate of Complications

Wound dehiscence	8 (14.0%)
	7 in unilateral cleft lip and palate
	1 in bilateral cleft lip and palate
Reoperation for dehiscence	3 (5.3%)
	all in unilateral cleft lip and palate
Postoperative infection	3 (5.3%)

## DISCUSSION

**Figure 8:**
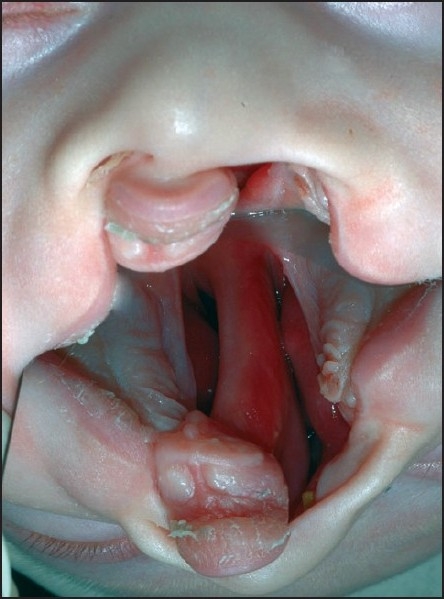
A patient with bilateral cleft lip and palate, with complete left lip cleftand incomplete right lip cleft, showing reduced extent of dysmorphology in incomplete clefts

The dysmorphology of incomplete clefts is reduced due to the presence of Simonart's band, which acts as a restrainer in utero, and reduces distortion [Figure 8].[6,15–16] Definitive lip repair achieves a more balanced lip and nose in incomplete clefts than complete ones.[2–6]

Based on these principles, Simon[17] and Hullihen[18] were the first to use lip adhesion before definitive closure. Later, Millard and Latham[3] and Spina[19] mentioned the use of lip adhesion in bilateral clefts of exceptional severity. Randall used lip adhesion in all patients with complete clefts to facilitate closure.[4]

The effect of soft tissue mobilisation on maxillary growth should not be underestimated.[6,20] Untreated patients with cleft lip show little growth disturbance, in contrast to those who have had surgery.[21–23] Extensive soft tissue undermining can have deleterious effects on maxillary growth.[6,24–25] Hence, a preliminary procedure that reduces the cleft size will allow a definitive repair with less undermining and less risk for growth disturbances.

Various nonsurgical adhesion methods have been introduced.[26–33] Preoperative lip taping is still in use.[6,34] Nonsurgical lip adhesion prevents an additional operation, but it requires compliance from the parents, with regular and costly in-office visits. Nonsurgical adhesion can result in epidermal stripping once the adhesive tapes are removed,[35] and its effect is uncontrollable without the concomitant use of presurgical orthopaedics.[6]

The controversy about presurgical orthopaedics is still ongoing. Different passive or active appliances have been described.[20,36] Burstone pioneered passive plating in cleft patients.[37] Surgery was postponed with the use of early preoperative guidance with a passive palatal plate, to allow for moulding with less growth impediment.[38–42] This therapy was intended to normalise feeding and tongue posture, to guide maxillary growth to normalcy, to support speech development and facilitate lip closure.[39,41–43]

Active presurgical orthopaedics was first popularized by McNeil,[32] then refined by Georgiade *et al*[44] and by Latham,[45–46] whose name became an eponym for active presurgical orthopaedics.[47]

Long-term results of POPLA (presurgical orthopaedics followed by periosteoplasty and lip adhesion) showed adverse effects on maxillary growth. While in bilateral clefts arch form, occlusion, and midfacial growth were considered acceptable, in most unilateral clefts anterior and lateral cross-bites were found at the age of six years.[48] Furthermore, more anterior open bites and posterior cross-bites were found in cleft patients treated with the POPLA procedure than in the control group, operated on by the same surgeon but without gingivoperiosteoplasty and active presurgical orthopaedics.[49] On the other hand, a multicenter, randomized clinical trial showed that presurgical orthopaedics realigned maxillary segments, diminishing anterior cleft width.[50]

Lip adhesion was advocated only in patients with bilateral clefts of exceptional severity[2–3,8,51–53] and also only in patients with unilateral clefts.[1,4,14,54–58] Lip adhesion, as part of the POPLA procedure, was implied in both unilateral and bilateral clefts patients.[3] We advocate lip adhesion in all patients with complete cleft lip, both unilateral and bilateral, with cleft palate or occasionally with P1 cleft of exceptional severity.

While in 1867 cleft lips were repaired a few hours after the baby's birth,[26] the suggested age of the infants at the time of lip adhesion ranges from two to five days until 3.5 months nowadays.[1,3,7–8,14,18,51–54,56–57] In our series, lip adhesion was performed at an average age of five weeks. This allows time for the infants to gain weight, for paediatricians to rule out concomitant malformations or diseases, and for parents to prepare for the burden of cleft children care.

There is a high degree of individualism in the technique of lip adhesion.[1,3,4,8,14,51–53,56–59] Marginal excisions of the cleft edges and interdigitation behind the prolabium, triangular mucosal flaps, and short, broad-based rectangular ones were recommended.[4,7,19] The C-W technique designed flaps from the vermilion.[59] Millard and Latham advocated a high, half-undermined adhesion, combining lip adhesion with periosteoplasty with incisions along the edges of the cleft.[3] This technique was applied without modification by some.[51–53,57–58]

There is agreement on the importance of muscle approximation during lip adhesion.[1,3–5,8,51–54,57–59] Muscle pull is essential for the moulding effect on maxillary arch segments.[16] Some addressed nasal deformity during lip adhesion and coined the term, the lip-nasal adhesion, to describe the procedure.[3,56] Dissection between the medial crura and alar cartilage,[56] dissection of columella, alar bases and the nasal septum, and nasal floor reconstruction were advocated.[3,51–53,57–58] We place the adhesion high, using structures that are discarded at definitive closure. Three-layer closure is achieved, approximating mucosa, muscle, and skin. Undermining of the lateral segment is minimized but allowed to facilitate tension-free closure. The nasal cartilages are not dissected, but severe septal deviation is corrected.

The timing of definitive closure after lip adhesion remains controversial. Definitive closure was performed six to 12 weeks,[59] three to four months,[1,8,56] and six months[7] after lip adhesion. Millard and Latham[3] and Cho[57–58] performed definitive repair at five to six months of age. Our definitive lip repair was performed at an average age of five months.

Presurgical orthopaedics combined with lip adhesion is an accepted method of early cleft management.[20] While presurgical orthopaedics means active segment management for many,[3,51–53,57,58] there are advocates of passive arch management with a palatal plate in combination with the lip adhesion.[10,11,60] To the best of our knowledge, the first report of successful application of lip adhesion with a passive guidance plate is from the Bruges Cleft and Craniofacial Centre.[10–11]

The opponents of lip adhesion emphasise wound dehiscence, requiring reoperation, and scar formation, destroying tissue valuable for definitive repair as major complications.[9] Long-term follow-ups do not mention these problems, reporting only sporadic midfacial retrusion with Angle Class III malformation.[3,51–52] Dehiscence was reported in 24% of bilateral clefts and 8% of unilateral cases in one study[7] and in 4% to 5% in unilateral clefts in another.[1] Scars in tissues to be used in the definitive repair posed no significant problem.[7] No postoperative haemorrhage or infection was seen.[1] In our series, 14% of the patients had postoperative partial or total dehiscence, but only those with total dehiscence (5.3%) needed reoperation. There was no scar formation that could interfere with definitive closure.

## CONCLUSION

Lip adhesion converts a complete cleft lip into an incomplete one, using tissue discarded at definitive repair. We used the combination of lip adhesion and guidance plate, creating an anatomical and functional background for definitive closure and primary rhinoplasty. Although complications such as infection and dehiscence occurred, the beneficial effects of lip adhesion outweighed those complications. Our surgical protocol addressed unilateral and bilateral cleft lips successfully.
